# Who Needs Cream and Sugar When There Is Eco-Labeling? Taste and Willingness to Pay for “Eco-Friendly” Coffee

**DOI:** 10.1371/journal.pone.0080719

**Published:** 2013-12-04

**Authors:** Patrik Sörqvist, Daniel Hedblom, Mattias Holmgren, Andreas Haga, Linda Langeborg, Anatole Nöstl, Jonas Kågström

**Affiliations:** 1 Department of Building, Energy and Environmental Engineering, University of Gävle, Gävle, Sweden; 2 Linnaeus Centre for Research on Hearing and Deafness, Swedish Institute for Disability Research, Linköping University, Linköping, Sweden; 3 Department of Economics, The University of Chicago, Chicago, Illinois, United States of America; 4 Research Institute of Industrial Economics, Stockholm, Sweden; 5 Department of Social Work and Psychology, University of Gävle, Gävle, Sweden; 6 Department of Business and Economic Studies, University of Gävle, Gävle, Sweden; University of Missouri-Kansas City, United States of America

## Abstract

Participants tasted two cups of coffee, decided which they preferred, and then rated each coffee. They were told (in lure) that one of the cups contained “eco-friendly” coffee while the other did not, although the two cups contained identical coffee. In Experiments 1 and 3, but not in Experiment 2, the participants were also told which cup contained which type of coffee before they tasted. The participants preferred the taste of, and were willing to pay more for, the “eco-friendly” coffee, at least those who scored high on a questionnaire on attitudes toward sustainable consumer behavior (Experiment 1). High sustainability consumers were also willing to pay more for “eco-friendly” coffee, even when they were told, *after* their decision, that they preferred the non-labeled alternative (Experiment 2). Moreover, the eco-label effect does not appear to be a consequence of social desirability, as participants were just as biased when reporting the taste estimates and willingness to pay anonymously (Experiment 3). Eco labels not only promote a willingness to pay more for the product but also lead to a more favorable perceptual experience of it.

## Introduction

An increasingly large number of products are marked with morally loaded labels such as ‘fair-trade’ and ‘organically produced’ – labels associated with social or environmental responsibility that speak to our conscience. ‘Moral’ labeling serves as a marketing device for attracting consumers with preference for social fairness or environmental altruism and some individuals are indeed willing to pay a premium for labeled products [1]–[4]. In this paper, we show that eco labels not only promote a willingness to pay more for the product but they also appear to enhance the perceptual experience of the product's taste.

Traditional analyses of consumer demand typically assume that individuals have well-defined preferences over product characteristics [5]. The perception of the product, such as flavor, does have a well-documented effect on consumers' willingness to pay [6]–[9]. However, perception is not a simple result of bottom-up sensory registration. Rather, it is modulated by top-down cognitive factors such as informational framing [10], [11] and expectations [12], [13]. For instance, adding a disgusting ingredient makes beer taste worse if people are told about it before tasting in comparison with uninformed consumers [14]. Information before tasting can also influence taste preferences of products that people believe differ from other products although they are actually objectively identical. For example, hamburgers [15], sodas [16] and beer [17] tend to taste better when their brand name is revealed (as long as the brand is associated with something positive), nutrition bars taste worse if the consumer falsely believe they contain soy [18], and wines taste better if people believe they are expensive [19]. Notably, these effects distort the basic sensory and perceptual experience rather than biasing self-reported taste preferences [20] and are particularly strong when objective taste is ambiguous [21].

Attitudes, like environmental concern, can also influence sensory ratings of the product. For instance, people with a positive attitude toward low-fat milk rate a taste-sample more positively when told about its low-fat character [22]. Moreover, people who endorse the values that a product symbolizes tend to rate the taste of the product more favorably [23], at least when the label is familiar [24]. Attitudes also influence decision making and may bias people's product choices toward eco-friendly alternatives [25]–[29]. By this logic, we hypothesized that if people have to make a taste preference decision between two products that are objectively identical but called ‘eco-friendly’ and ‘not eco-friendly’ respectively, they will tend to choose the ‘eco-friendly’ alternative, at least individuals for whom an eco-friendly label has positive associations, especially because the taste difference between the two objectively identical products are clearly ambiguous in this situation, and the preference judgment should therefore be particularly susceptible to expectations and stereotypical believes (cf. [21]).

## Experiment 1

Experiment 1 was designed to test whether participants tend to prefer the taste of, and be willing to pay a higher price for, ‘eco-friendly’ coffee in relation to ‘not eco-friendly’ coffee (even though the two cups of coffee they taste are objectively identical). A more specific hypothesis was that the tendency to demonstrate a preference for the ‘eco-friendly’ alternative would vary with the participants' attitudes. Participants who report positive attitudes toward sustainable consumer behavior (i.e., buy eco-friendly products, pre-separate waste at source, and feel guilt when not buying eco-friendly alternatives) should be more biased toward the ‘eco-friendly’ alternative. Participants were requested to taste two separate cups of coffee. Unbeknownst to the participants, the two cups contained coffee from the exact same brew and brand. Before tasting, they were told (in lure) that one of the cups contained ‘eco-friendly’ coffee and that the other cup did not and they were told which of the two cups that contained the ‘eco-friendly’ alternative.

### Methods

#### Participants

A total of 44 individuals (mean age  = 27.71 years, *SD*  = 12.22) were recruited to participate in Experiment 1. Based on a questionnaire (see below), they were classified as ‘high sustainability’ consumers (*N* = 23) or ‘low sustainability’ consumers (*N* = 21) respectively. This study was approved by the Uppsala regional ethical review board (Dnr 2013/132). As the data was treated confidentially, and no apparent ethical research complication with participation could be identified, oral consent was deemed sufficient by the ethical review board. The data collectors took note of the oral consent.

#### Materials

*Coffee.* The to-be-tasted coffee was brewed on milled *coffea arabica* beans, using a standard model coffee machine. The just-made coffee was poured into a thermos to maintain heat during the data collection.

*Questionnaire.* A questionnaire was used to obtain data. First, the participants circled which of the two coffees just tasted that they liked the most (the ‘eco-friendly’ or the ‘not eco-friendly’). On the next page, the first questions were “On a scale from 1–7, what did you think of the taste of the ecological/non-ecological coffee?”. The participants were requested to circle the number (1–7 with the endpoints labeled “Not at all tasty” and “Very tasty” respectively) that would best describe their taste evaluation. The next question was “How much would you be willing to pay for a package of the coffee you just tasted? (The mean price of a package of coffee is about 45 Swedish Kronor)”. The participants marked their responses for the ‘eco-friendly’ and for the ‘not eco-friendly’ coffee, respectively. The order between the to-be-rated coffees (‘eco-friendly’ first versus ‘not eco-friendly’ first) was counterbalanced between participants. Half received a questionnaire in which questions concerning the ‘eco-friendly’ coffee were presented first. The order was reversed for the other half of the participants. On the final pages of the questionnaire, the participants were asked to report age, number of children, number of persons in the household, the total household income, and how many cups of coffee they drink per day on average. They were also asked to respond to the following questions, on a scale from 1–7 (endpoints labeled): “How often do you buy eco-labeled products?”, “How important is it to you to buy eco-friendly alternatives?”, “Do you pre-separate your waste at source?”, and “Do you feel guilt when you buy non-eco-friendly alternatives?”. The responses across the four questions were averaged to create an index of attitudes toward sustainable consumption. Participants were classified as high or low sustainability consumers based on whether their value was above or below the median index score. Participants who had a score equal to the median were classified based on whether they were above or below the mean on the question that concerned guilt.

#### Design and procedure

The experiment took place in a sealed cubicle in a corridor at a university campus. People passing by the cubicle were recruited as participants. Just before the participant entered the cubicle, the test leader poured coffee from the thermos into two separate cups. The participants’ task was to (1) taste the coffee from both cups, (2) answer the question “which one did you like the best?”, and (3) fill in the questionnaire. The participants drank a mouthful of tap water between the two cups of coffee. The test leader told the participants, verbally, before they tasted, that one cup contained ‘eco-friendly’ coffee and that the other cup did not (although the two cups of coffee were objectively identical). They were also told which of the two cups that contained ‘eco-friendly’ coffee. The order of tasting for the two cups of coffee was counterbalanced between participants, so that half tasted the ‘eco-labeled’ coffee first and the other half tasted the ‘not eco-friendly’ coffee first.

### Results

Among all participants, 27 choose the ‘eco-friendly’ coffee and 17 choose the ‘not eco-friendly’ coffee in the forced choice task. Mean sustainable consumer behavior index was 3.85 (*SD*  = 1.28, median  = 4) and Cronbach's alpha was .81. Higher index scores were associated with a greater tendency to choose the ‘eco-friendly’ coffee, as shown in a logistic regression analysis, *B* = .70, *SE*  = .29, *p* = .016. Higher index scores were also associated with a greater taste preference for the ‘eco-friendly’ coffee (difference score between the ‘eco-friendly’ and the ‘not eco-friendly’ alternative; *M* = 0.23, *SD*  = 1.80), *r*(42)  = .28, *p* = .032 (one-tailed), and with a greater willingness to pay a premium for the ‘eco-friendly’ coffee (difference score between the ‘eco-friendly’ and the ‘not eco-friendly’ alternative; *M* = 4.34, *SD*  = 10.57), *r*(42)  = .53, *p*<.001. Overall, greater taste preference was associated with a higher willingness to pay, as indicated by a highly significant positive correlation between the difference scores for taste ratings and for willingness to pay, *r*(42)  = .56, *p*<.001. Sustainable consumer behavior index was not significantly related to any of the demographic variables.

#### Group differences in taste ratings

**Figure 1:**
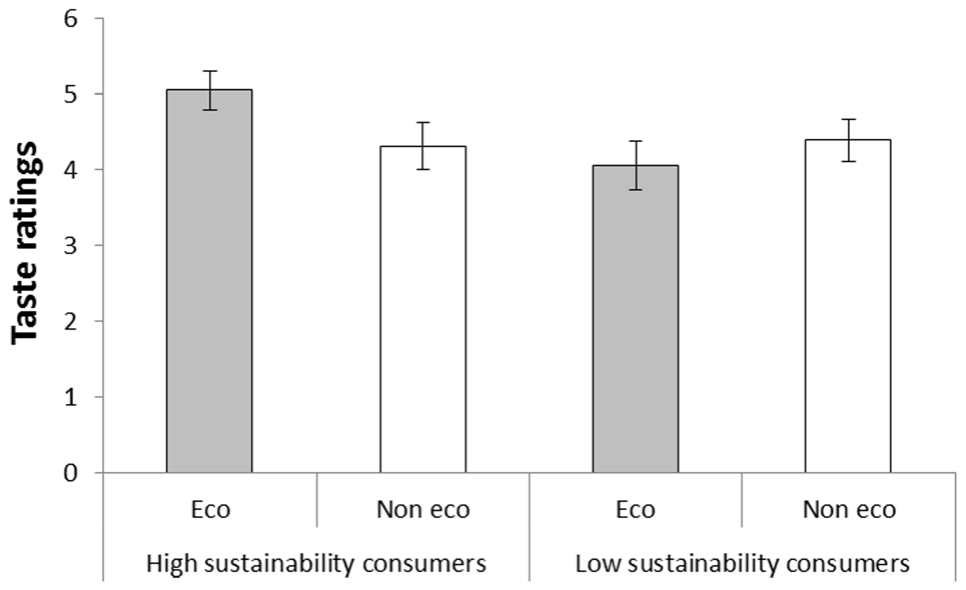
Taste ratings of coffee called ‘eco-friendly’ and ‘not eco-friendly’, respectively, by participants classified as high and low sustainability consumers in Experiment 1. Error bars represent standard error of means.

To simplify cross-experiment comparisons and result interpretations, additional analyses with participants dichotomized into high and low sustainability consumers, as described in the methods section, are reported. In the forced choice task, 74% of the high sustainability consumers choose the ‘eco-labeled’ coffee and 26% choose the ‘not eco-friendly’ coffee, whereas 48% of the low sustainability consumers choose the ‘eco-labeled’ coffee and 52% choose the ‘not eco-friendly’ alternative. As can be seen in Figure 1, the high sustainability consumers demonstrated a slight taste preference for the ‘eco-labeled’ coffee, even though there was no objective difference between the two cups of coffee. The low sustainability consumers, on the other hand, demonstrated no preference for either coffee. This conclusion was supported by a 2(Label: eco-friendly vs. not eco-friendly) ×2(Group: high vs. low sustainability) analysis of variance with taste ratings as dependent variable. The analysis revealed no significant main effect of label, *F*(1, 42)  = 0.60, *MSE*  = 0.90, *p* = .444, η_p_^2^ = .01, and no significant effect of group, *F*(1, 42)  = 2.11, *MSE*  = 2.20, *p* = .153, η_p_^2^ = .05, but a significant interaction between the two factors, *F*(1, 42)  = 4.17, *MSE*  = 1.51, *p* = .047, η_p_^2^ = .09. High sustainability consumers demonstrated a significant taste preference for the coffee labeled ‘eco-friendly’, *t*(22)  = 2.10, *p* = .047.

#### Group differences in willingness to pay

**Figure 2:**
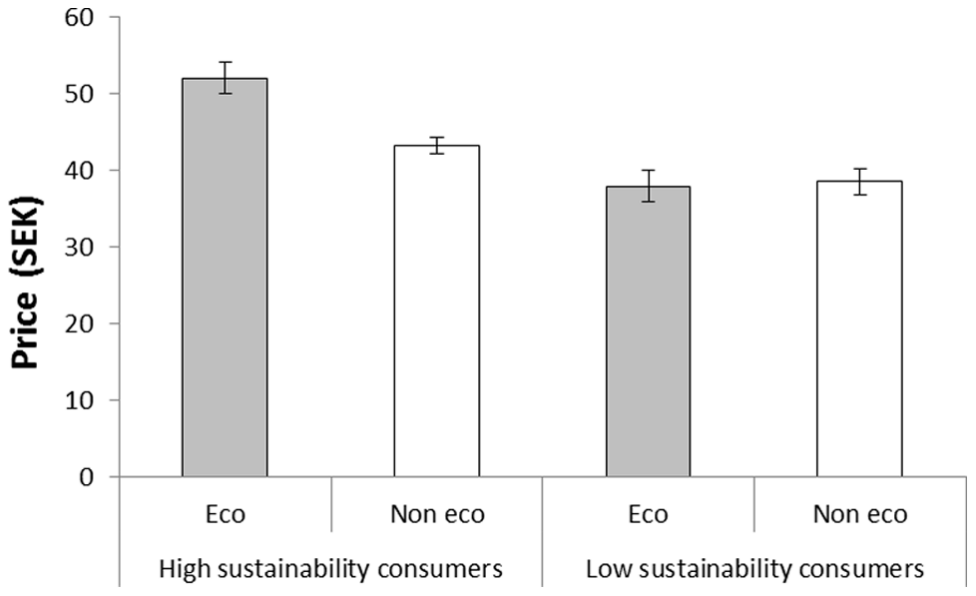
The price participants (classified as high and low sustainability consumers) were willing to pay for coffee called ‘eco-friendly’ and ‘not eco-friendly’, respectively, in Experiment 1. Error bars represent standard error of means.

As can be seen in Figure 2, the high sustainability consumers were willing to pay a larger premium for the ‘eco-friendly’ coffee in comparison with the low sustainability consumers. This conclusion was supported by a 2(Label: eco-friendly vs. not eco-friendly) ×2(Group: high vs. low sustainability) analysis of variance with willingness to pay as dependent variable. The analysis revealed a significant main effect of label, *F*(1, 42)  = 8.20, *MSE*  = 45.60, *p* = .007, η_p_^2^ = .16, a significant effect of group, *F*(1, 42)  = 21.43, *MSE*  = 90.53, *p*<.001, η_p_^2^ = .34, and a significant interaction between the two factors, *F*(1, 42)  = 10.63, *MSE*  = 45.60, *p* = .002, η_p_^2^ = .20. High sustainability consumers demonstrated a significant willingness to pay a premium for the eco-friendly alternative, *t*(22)  = 6.36, *p*<.001, but the low sustainability consumers did not, *t*(22)  = −0.22, *p* = .829.

### Discussion

It has been shown that people prefer the taste of organically produced products [27], [30]. If there is an objective difference between organically produced and conventional products, this may not be surprising. Yet, we have shown that, with the right convictions, an ‘eco-friendly’ *label* is sufficient for a product to taste better than a non-labeled objectively identical alternative. This eco-label effect suggests that top-down expectation processes influence taste perception.

Previous research indicates that consumers must perceive high quality in order for a food product to command a premium [9] and that consumers are not prepared to pay extra for the sake of environment alone [31]. This is consistent with the high positive correlation between taste and willingness to pay ratings reported in Experiment 1. One possibility is that high sustainability consumers actually report a higher willingness to pay for the ‘eco-friendly’ coffee because they think it tastes better, not necessarily because they wish to make a sustainable consumer decision (i.e., for more altruistic reasons). However, some consumers are still willing to pay a premium for eco-friendly products when told that the products have no personal benefits [32], a result that indicates that willingness to pay the premium is not entirely underpinned by self-serving reasons (e.g., better taste). Experiment 2 was designed to address this question by tearing apart the co-variation between sustainability attitudes, taste preference and willingness to pay.

## Experiment 2

In Experiment 2, participants were requested to taste coffee from two different cups as in Experiment 1, but this time they were not told which one of the two cups that contained eco-friendly coffee until after they made the preference decision. After their decision, half of the participants were told that their preferred coffee was eco-friendly (*eco-preference condition*) and the other half was told that they preferred the coffee that was not eco-friendly (*non-eco-preference condition*). If high sustainability consumers base their willingness to pay a premium for the eco-friendly coffee on biased taste preferences, they should be unwilling to pay a premium for it when told that they prefer the taste of the non-labeled alternative, but if it is based on care for the environment, they should still be willing to pay the premium for eco-friendly coffee when they prefer the taste of the non-labeled coffee. The willingness to pay of low sustainability consumers, who arguably are indifferent to the label, should be more consistent with their taste preferences.

### Methods

#### Participants

A total of 87 individuals (mean age  = 28.08 years, *SD*  = 10.56) were recruited to participate in Experiment 2. There were 20 high sustainability consumers and 23 low sustainability consumers in the eco-preference condition and 21 high sustainability consumers and 23 low sustainability consumers in the non-eco-preference condition.

#### Materials

The materials, coffee and questionnaire were identical to Experiment 1.

#### Design and procedure

The design and procedure were identical to Experiment 1 with the exceptions noted. The test leader told the participants verbally before they tasted that one cup contained ‘eco-friendly’ coffee and that the other cup did not, but they were not told which of the two cups that was supposed to contain ‘eco-friendly’ coffee until after they indicated which of the two cups of coffee they preferred (but before they made taste and willingness to pay ratings). The participants were randomly assigned to two conditions. Half were told that the coffee they preferred was ‘eco-friendly’ (eco-preference condition) and the other half was told that they preferred the ‘not eco-friendly’ alternative (non-eco-preference condition).

### Results

Mean sustainable consumer behavior index was 3.89 (*SD*  = 1.10, median  = 4) and Cronbach's alpha was .69. Index scores were unrelated to the demographic variables except for number of children, *r*(85)  = .25, *p* = .018.

#### Group differences in taste ratings

**Figure 3:**
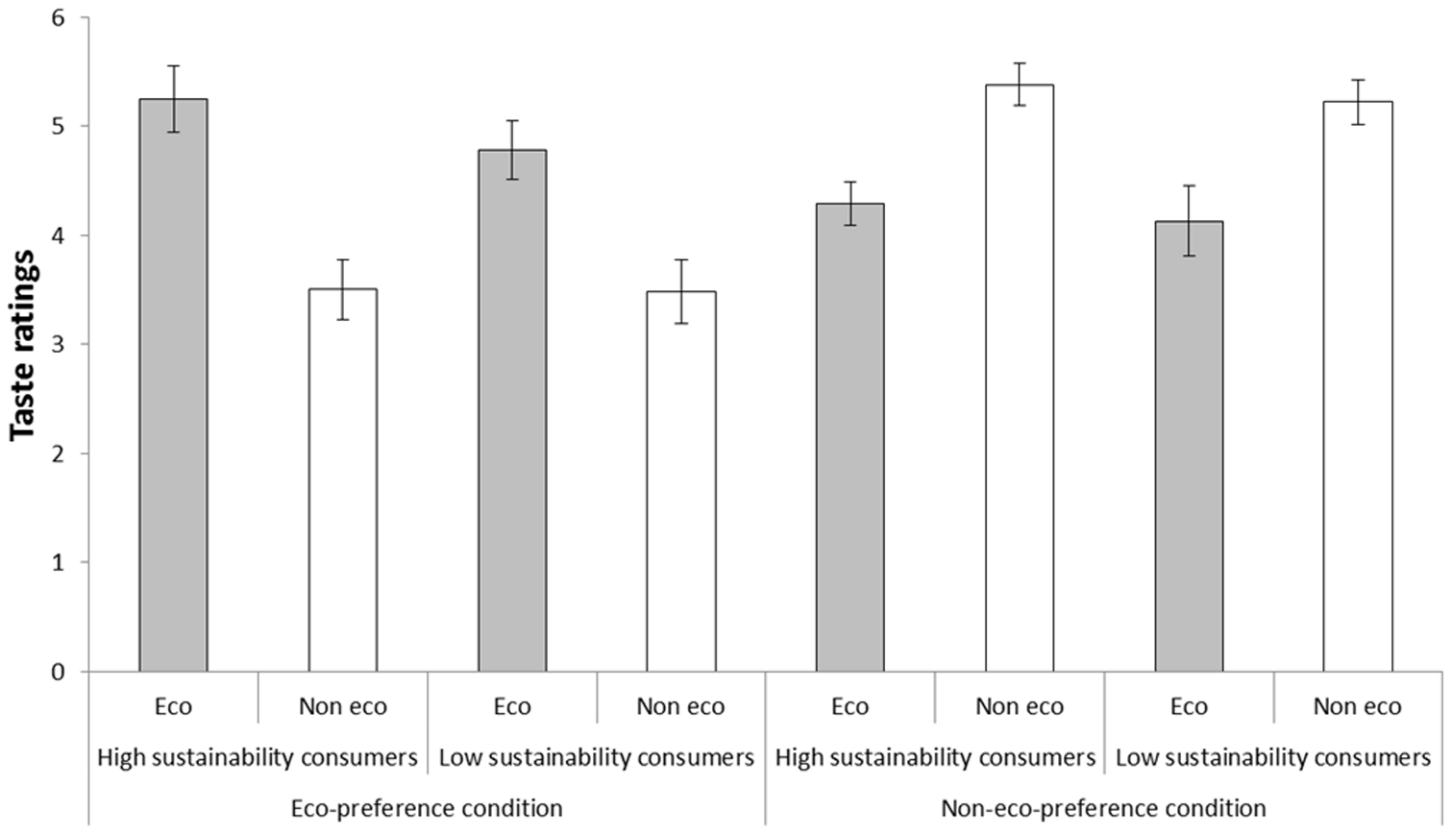
Taste ratings of coffee called ‘eco-friendly’ and ‘not eco-friendly’, respectively, by participants classified as high and low sustainability consumers in Experiment 2, who either took part in an eco-preference condition wherein participants were told that their preferred coffee is the ‘eco-friendly’ alternative or in a non-eco-preference condition wherein participants were told that their preferred coffee is the ‘not eco-friendly’ alternative. Error bars represent standard error of means.

In this experiment, taste ratings served as a control of whether the experimental manipulation had been successful. Figure 3 shows that the participants in the two conditions reported taste ratings consistent with the condition to which they had been assigned. Participants in the eco-preference condition reported higher taste ratings for the ‘eco-friendly’ coffee and participants in the non-eco-preference condition reported higher taste ratings for the ‘not eco-friendly’ alternative, regardless of their attitudinal predispositions. Most notably, high sustainability consumers in the non-eco-preference condition reported a significant taste preference for the ‘not eco-friendly’ coffee, *t*(20)  = 5.32, *p*<.001.

#### Group differences in willingness to pay

**Figure 4:**
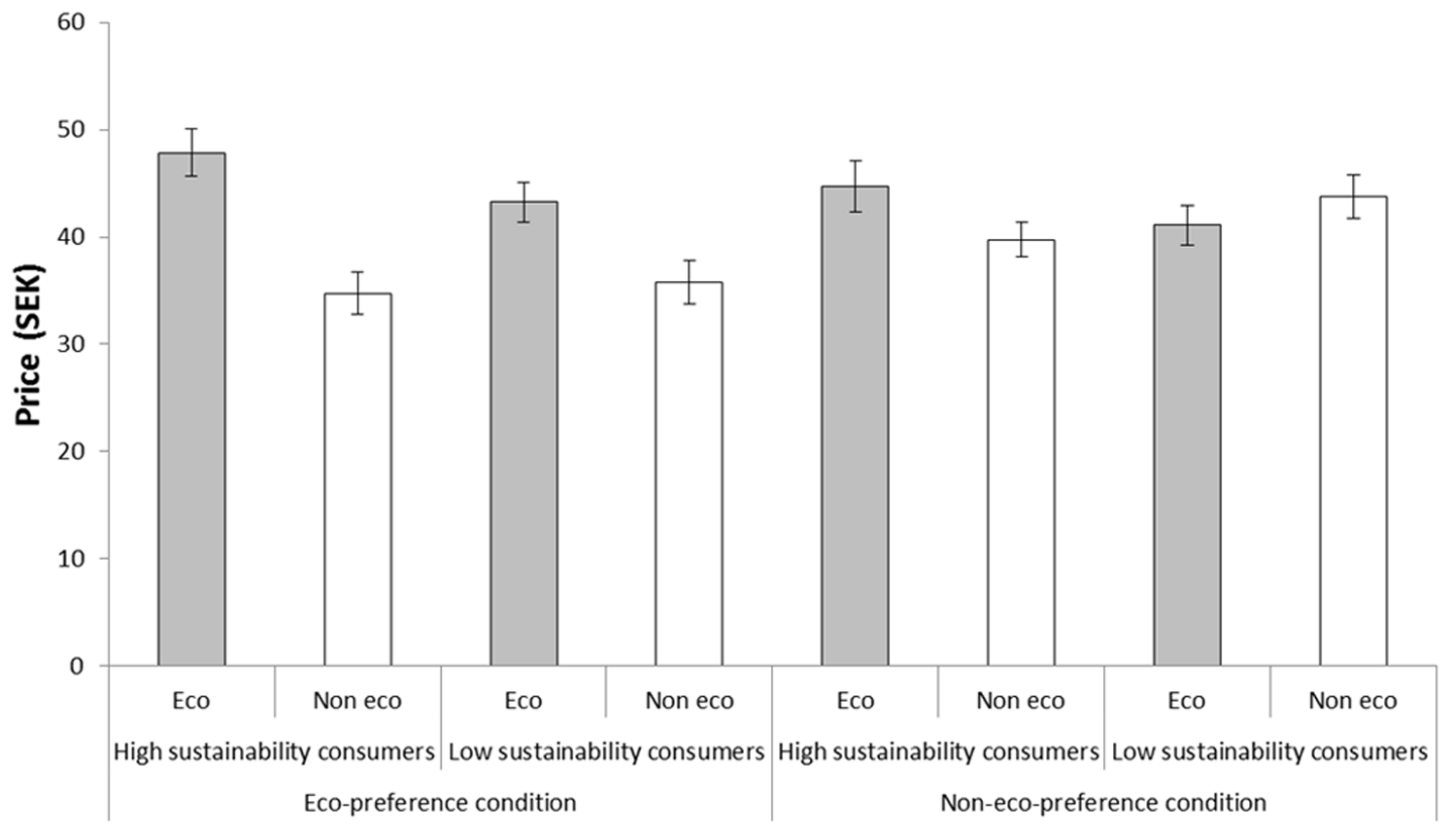
The price participants (classified as high and low sustainability consumers) were willing to pay for coffee called ‘eco-friendly’ and ‘not eco-friendly’, respectively, in Experiment 2. The participants either took part in an eco-preference condition wherein they were told that their preferred coffee is the ‘eco-friendly’ alternative or in a non-eco-preference condition wherein they were told that their preferred coffee is the ‘not eco-friendly’ alternative. Error bars represent standard error of means.

High sustainability consumers in the non-eco-preference condition, who reported a significant taste preference for the ‘not eco-friendly’ coffee (Figure 3), were still prepared to pay a premium for the ‘eco-friendly’ alternative, *t*(20)  = 2.53, *p* = .020 (Figure 4). However, this premium was statistically smaller than the premium high sustainability consumers in the eco-preference condition were willing to pay, *t*(39)  = 2.91, *p* = .006. Low sustainability participants in the eco-preference condition were also willing to pay a premium for the ‘eco-friendly’ coffee, *t*(22)  = 5.47, *p*<.001. The low sustainability consumers in the non-eco-preference condition were, on average, not willing to pay a statistically significant premium for the ‘not eco-friendly’ coffee, *t*(22)  = 1.52, *p* = .142.

### Discussion

Low sustainability consumers appear to be willing to pay more for the eco-friendly alternative as long as they prefer the taste of that product. When they prefer the taste of a non-labeled product, they still seem unwilling to make the morally stigmatizing decision of paying more for the non-labeled alternative. In contrast, high sustainability consumers are willing to pay a higher price for eco-friendly products even when they believe that they prefer the taste of a non-labeled alternative. This finding indicates that their willingness to pay a premium for eco-friendly alternatives is – at least in part – based on altruistic (e.g., for the sake of the environment) rather than on more self-serving reasons (e.g., biased taste preferences). However, they do seem to consider taste when deciding for a product price, as the premium they are willing to pay for the eco-friendly alternative, when they prefer the taste of a non-labeled alternative, is significantly smaller than when they prefer the taste of the eco-friendly alternative. Together, these results indicate that willingness to pay is in part based on the eco-label and in part on the taste ratings.

An alternative interpretation of the results up till this point is that the participants – at least the high sustainability consumers – felt a need to conform to social desirability (i.e., the tendency of respondents to adapt their behavior and decisions to be viewed favorably by others) and therefore reported more favorable ratings of the eco-friendly coffee. This possibility is potentiated as pro-environment behavior and attitudes are normative [33]–[35]. Although ethical convictions tend to motivate consumers' choice of organic/eco-friendly food [36], social desirability seems to influence willingness to pay for eco-friendly products [37]–[39]. Experiment 3 was designed to test whether social desirability underpins the eco-preference bias found in Experiments 1 and 2.

## Experiment 3

In Experiment 3, the experimental design of Experiment 1 (in which the participants were told about the coffee's label before tasting) was revisited, but this time the participants were randomly assigned to either of two experimental conditions. In one of the conditions (*revealed ratings condition*), the participants made their ratings by reporting them to the experimenter, and the experimenter noted their responses on a response sheet. In the other experimental condition (*concealed ratings condition*), the experimenter left the room before the ratings were made, the participants noted their ratings on the response sheet themselves and slipped the sheet into a sealed box before leaving the room. We hypothesized that if social desirability underpins the eco-label effect on taste and willingness to pay, there should be a systematic difference between the conditions. In turn, if expectations and positive associations toward the eco-label underpin the eco-label effect rather than social desirability, there should be no difference between the conditions.

Experiment 3 also served a secondary purpose. If positive associations underpin the eco-label effect on taste perception, the effect should be stronger within participants who claim that eco-labeled products generally taste better than non-labeled alternatives. To test this hypothesis, we asked the participants about their general taste preference for eco-labeled and non-labeled products.

### Methods

#### Participants

A total of 40 individuals (mean age  = 24.75 years, *SD*  = 3.46) were recruited to participate in Experiment 3.

### Materials

The materials were identical to those in Experiment 1 with one exception. The question “Which one do you prefer the taste of generally?” was added at the end of the questionnaire. The scale 1–7 was used for answering. The lowest value (i.e., 1) was labeled “eco-labeled products”, the middle value (i.e., 4) was labeled “same” and the highest value (i.e., 7) was labeled “non-labeled products.

#### Design and procedure

The design and procedure was identical to Experiment 1 with the exceptions noted. The experiment took place in an isolated laboratory room. The participants were randomly assigned to either of two conditions. After tasting the two cups of coffee, half of the participants were requested to report the taste ratings and what they were willing to pay for a package of each type of coffee to the experimenter, and the experimented noted their responses on the response sheet (revealed ratings condition). For the other half (concealed ratings condition), the experimenter left the room before the ratings were made, the participants noted the ratings on the response sheet themselves and slipped the sheet into a sealed box before leaving the room. All participants were later asked to enter a different laboratory room to fill in the questionnaire. They were all alone when filling in the questionnaire.

### Results

#### Taste

**Figure 5:**
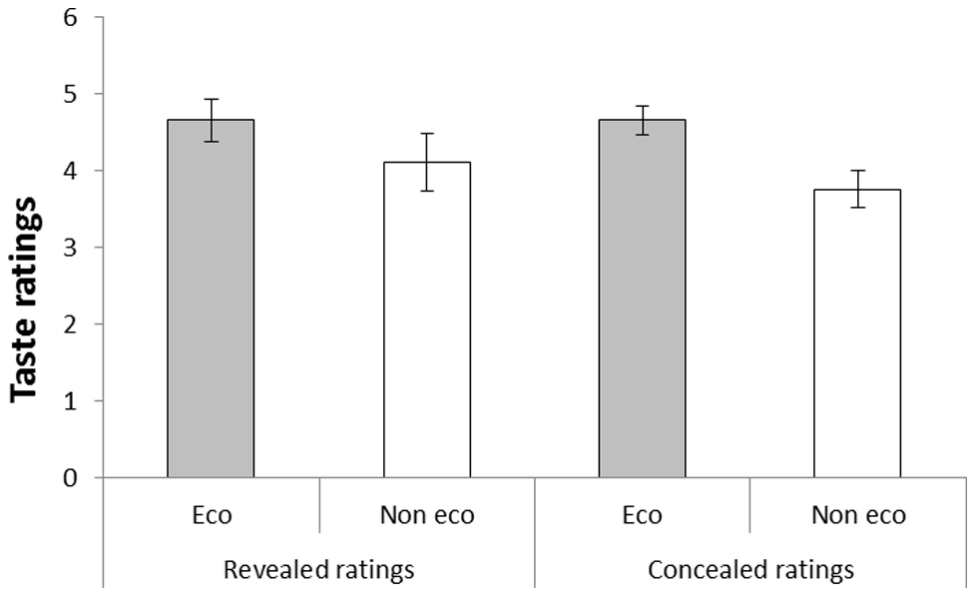
Taste ratings of coffee called ‘eco-friendly’ and ‘not eco-friendly’, respectively, in Experiment 3. The participants either reported their ratings to the experimenter (revealed ratings condition) or made their ratings anonymously (concealed ratings condition). Error bars represent standard error of means.

As can be seen in Figure 5, the participants in both conditions preferred the taste of the coffee that had arbitrarily been assigned an ‘eco-friendly’ label over an objectively identical non-labeled alternative. However, there was no difference between the conditions. These conclusions were supported by a 2(Condition: revealed vs. concealed reports) ×2(Label: eco-friendly vs. not eco-friendly) analysis of variance with taste ratings as dependent variable. There was a significant main effect of label, *F*(1, 38)  = 9.66, *MSE*  = 1.09, *p* = .004, η^2^_p_ = .20, but no significant main effect of condition, *F*(1, 38)  = 0.23, *MSE*  = 2.67, *p* = .634, η^2^_p_ = .01, and no interaction between the factors, *F*(1, 38)  = 0.56, *MSE*  = 1.09, *p* = .458, η^2^_p_ = .02.

The non-significant effect of condition and the non-significant interaction indicate that social desirability does not influence the eco-label effect on taste. However, this conclusion rests on an affirmed null-hypothesis, which is problematic in the context of conventional inferential statistics. Because of this, we also report the Bayes factors for the main effects and the interaction, as Bayesian statistics can support the null [40]. The main advantage of Bayesian analysis compared to the conventional frequentists' approach is that Bayesian statistics provide a tool to calculate the actual probability of a hypothesis given the data (i.e., *p*(H_0_|D)) instead of calculating the probability of the observed data (i.e., *p*(D|H_0_)) under the assumption that H_0_ is true. Bayes factors are calculated by dividing the probability for the null hypothesis (H_0_) with the probability for the alternative (H_1_). If this value is less than 1, the evidence favors the alternative, and if the value is greater than 1, the evidence instead favors H_0_. The Bayes factor was 0.07 (very strong evidence for H_1_) for the effect of label on taste, 5.46 (positive evidence for H_0_) for the effect of condition on taste, and 4.72 (positive evidence for H_0_) for the interaction between the label and condition. See a paper by Raftery [41] for thresholds for whether the evidence supports the null-hypothesis or the alternative.

#### Willingness to pay

**Figure 6:**
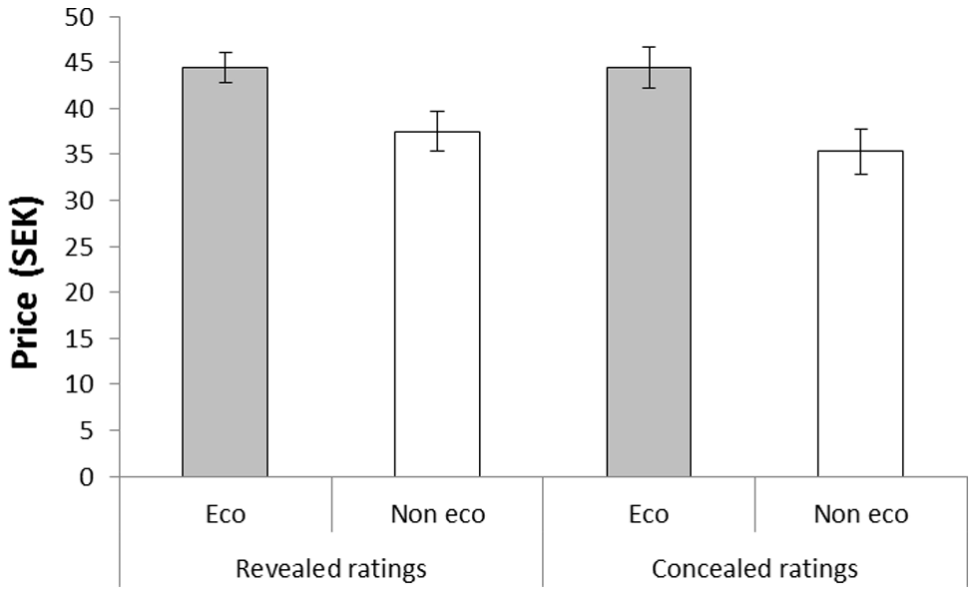
Willingness to pay for coffee called ‘eco-friendly’ and ‘not eco-friendly’, respectively, by participants who either reported their ratings to the experimenter (revealed ratings condition) or who made their ratings anonymously (concealed ratings condition) in Experiment 3. Error bars represent standard error of means.

Figure 6 shows that the participants in both conditions were willing to pay more for the coffee that had arbitrarily been assigned an ‘eco-friendly’ label over an objectively identical non-labeled alternative. Again, there was no difference between the conditions. These conclusions were supported by a 2(Condition: revealed vs. concealed reports) ×2(Label: eco-friendly vs. not eco-friendly) analysis of variance with taste ratings as dependent variable. There was a significant main effect of label, *F*(1, 38)  = 30.05, *MSE*  = 43.13, *p*<.001, η^2^_p_ = .44, but no significant main effect of condition, *F*(1, 38)  = 0.16, *MSE*  = 147.54, *p* = .688, η^2^_p_ = .004, and no interaction between the factors, *F*(1, 38)  = 0.56, *MSE*  = 43.13, *p* = .458, η^2^_p_ = .02. Again, we report the Bayes factors for the analysis. The Bayes factor was 0.00005 (very strong evidence for H_1_) for the effect of label on willingness to pay, 5.80 (positive evidence for H_0_) for the effect of condition on willingness to pay, and 4.72 (positive evidence for H_0_) for the interaction between the label and condition.

#### Individual difference analyses

We first calculated the difference scores for taste ratings (mean taste difference  = 0.73, *SD*  = 1.47) and willingness to pay (mean willingness to pay difference  = 8.05, *SD*  = 9.23). A correlation analysis found that the magnitude of taste difference was positively related to the magnitude of willingness to pay difference, *r*(38)  = .60, *p*<.001. This suggests that the participants considered taste when they decided for a price. The individual difference measures were used to analyze whether consumers attitudinal predispositions influence the magnitude of the eco-label effect as in Experiment 1. In Experiment 3, mean sustainable consumer behavior index was 4.03 (*SD*  = 1.21, median  = 4) and the scale's Cronbach's alpha was .73. This time, higher index scores were not significantly related to the preference decisions in the forced-choice task, *B* = −.11, *SE*  = .28, *p* = .709. Furthermore, there was no relation between index scores and taste preference for the ‘eco-friendly’ coffee, *r*(38)  = .16, *p* = .328, or with willingness to pay a premium for the ‘eco-friendly’ coffee, *r*(38)  = .11, *p* = .491. The same conclusion held even if the correlations were analyzed separately for the experimental conditions.

The sample mean for the question concerning whether the participants generally prefer the taste of eco-labeled or non-labeled products, wherein lower values represented a general taste preference for eco-labeled products, was 3.37 (*SD*  = 1.01, range 1–4, possible range 1–7). Greater taste preference for eco-labeled products in general was associated with a greater taste preference for the ‘eco-friendly’ over the ‘not eco-friendly’ coffee, *r*(38)  = −.50, *p*<.001, and with a willingness to pay a greater premium for the ‘eco-friendly’ coffee, *r*(38)  = −.40, *p* = .010. Thus, the eco-label effect was greater in magnitude amongst individuals with a general taste preference for eco-friendly products. In other words, participants with an explicit positive attitude toward the general taste of eco-labeled products were more biased to believe that the ‘eco-friendly’ coffee tasted better than the objectively identical non-labeled coffee. Furthermore, a general taste preference for eco-labeled products was associated with a higher sustainability consumer index score, *r*(38)  = −.45, *p* = .004. Taken together, the results suggest that expectations concerning taste might be a stronger predictor of the magnitude of the eco-label effect than our sustainability consumer index.

### Discussion

Experiment 3 replicated the eco-label effect: Both taste and willingness to pay is biased towards a preference for eco-labeled coffee over an objectively identical non-labeled alternative. Moreover, it appears like the effect is not underpinned by social desirability, as the participants' taste and willingness to pay were just as biased when they made their ratings anonymously as when they reported the ratings directly to the experimenter. If anything, the eco-label effect was stronger (although the difference did not reach significance) when anonymous ratings were made. The relationships with sustainable consumer index were, however, not replicated in Experiment 3, which suggests that the eco-label effect on taste might be a more general phenomenon than we first anticipated (i.e., not manifested in high sustainability consumers only). The magnitude of the eco-label effect on taste appears rather to depend on participants general convictions about the taste difference between eco-labeled and conventional products.

## General Discussion

The series of experiments reported here revealed three main findings. First, there is an eco-label effect on taste and willingness to pay such that people are biased to prefer coffee that has been arbitrarily labeled ‘eco-friendly’ over an objectively identical non-labeled alternative; second, social desirability (i.e., the need to express believes and behaviors to gain the appeal of others) appears not to underpin the eco-label effect; and third, people who are willing to pay a premium for eco-friendly coffee does so even when they believe that they prefer the taste of a non-labeled alternative, at least people who score high on a sustainable consumer behavior scale. The results are discussed in detail below.

### Taste perception

Taste perception is sensitive to contextual factors like informational framing [11], labels [18] and expectations [12], which in turn are qualified by individual predispositions like attitudes [22], [24]. The results reported here extend this literature into the environmental domain by showing that people may be biased toward a taste preference for eco-labeled alternatives.

The eco-label effect was approximately equal in magnitude for the participants who made anonymous taste ratings (and willingness to pay ratings) and the participants who explicitly reported the ratings to the experimenter. This finding suggests that the participants did not report higher taste (and willingness to pay) ratings for the eco-friendly alternative at the influence of social desirability. Furthermore, it has been shown that labels create expectations that influence the actual sensory processing rather than having its impact on biased self-reported ratings [20]. Taken together, it appears as if social desirability does not underpin the eco-label effect.

The magnitude of the eco-label effect appears to depend on explicit attitudes. In Experiment 1, the eco-label effect was only present in participants who reported high values on a sustainable consumer behavior scale (e.g., participants who often buy eco-labeled products and feel guilt when not doing so). The eco-label effect was replicated in Experiment 3, but in this experiment it was not related to the sustainable consumer behavior scale. In turn, Experiment 3 found that a generally positive attitude toward the taste of eco-labeled products predicted the magnitude of the eco-label effect on taste. Taken together, we therefore favor the view that the eco-label actually modifies the taste perception in individuals for whom the label symbolizes something positive because it is intrinsically rather than socially desirable. Whether eco-labeled products taste better because of attitudes toward sustainable consumer behavior that influence taste expectations, or more specifically because of attitudes toward the taste of eco-labeled products, remains less clear.

One reason why an eco-friendly label influence taste perception could be that people have stereotypical believes about how the production process differs for eco-friendly and conventional products. In the case of crop products, like coffee, consumers could quite easily imagine production differences that could influence taste (e.g., crop-spraying). A potentially interesting research continuation along these lines would be to investigate whether an eco-label has a similar effect on taste preferences for other products (e.g., root beer) for which the participants may have difficulty imagining how the production process could differ in such a way that it would have an effect of taste qualities. Another interesting continuation would be to investigate whether other labels that signal social responsibility – like ‘fair trade’ – have similar effects on taste perception as the eco-label. Interestingly, people seem to believe that ‘fair trade’ products (e.g., chocolate) are healthier than non-labeled products [42]. One possibility is that morally loaded labels – like ‘fair trade’, ‘organic’ and ‘eco-friendly’ – have general halo effects favorably influencing subjective product characteristics across a range of judgmental dimensions.

### Willingness to pay

How do people respond when they are told, *after* making a preference decision, that their preferred coffee is the ‘morally’ righteous option (i.e., the eco-friendly alternative) vis-à-vis when they are told that their preferred alternative is the ‘immoral’ option? High sustainability consumers seem to be willing to pay more for a labeled product, even when they prefer the taste of a non-labeled alternative (Experiment 2). This finding is important because it shows that the eco-label effect on willingness to pay found in Experiments 1 and 3 is not simply reflecting the positive correlation between taste and willingness to pay. Rather, willingness to pay seems to be based on taste in addition to the eco-label. Other factors may also be involved. One possibility is that high sustainability consumers exhibit an effect analogous to the so-called *endowment effect* [43] when exposed to a ‘moral’ label. When the preferred alternative is revealed to be ‘not eco-friendly’, these individuals need to defend their decision by increasing the relative willingness to pay for the chosen (‘not eco-friendly’) alternative, but to maintain moral standard they are still willing to pay more for the eco-friendly alternative. The traditional endowment is the propensity of individuals to increase willingness to accept (i.e., the subjective value of the good) for an object with which they have been endowed, while at the same time decrease willingness to pay for another object. In our setting, the decision of taking stand on whether the alternative with a ‘moral’ label taste better than the non-labeled alternative increases the willingness to pay for the chosen alternative. Another possible explanation is that the high sustainability consumers experience *cognitive dissonance* (i.e., feelings of ‘inner stress’ as a result of inconsistent attitudes or behaviors; [44]) when they learn that they prefer the taste of the ‘immoral’ (i.e., the conventional rather than the eco-friendly) alternative. They adjust the size of the premium to be consistent with their taste preference, but as this is inconsistent with their self-image and leads to cognitive dissonance, they resolve the dissonance by still reporting a higher willingness to pay for the eco-friendly alternative [45].

Taken together, the results thus speak against the notion that consumers are not prepared to pay extra for the sake of the environment alone [31]. On the contrary, our findings are entirely consistent with the idea that people may view a morally loaded label as an additional characteristic of the good. The moral thing to do (i.e., buying an eco-friendly product even though it costs more than a conventional alternative) appears to be a more important determinant to some individuals than tangible product characteristics. This interpretation accords well with related studies suggesting that evoking images (such as that of eco-labeling) that appeal to the social responsibility of consumers can lead consumers to desired behaviors [46], that consumer behavior is related to self-image motives [47], [48] and that people sometimes make pro-environment decisions to establish a view of the self as a morally righteous individual [49], [50]. Another possibility, however, that is inconsistent with the idea that altruistic rather than egoistic reasons underpin the high sustainability consumers' behavior, is that they view the label as a signal of higher quality (e.g., that the product is healthier). This latter possibility is consistent with extant research suggesting that consumers must perceive a higher quality in order for a food product to command a premium [9], but it is inconsistent with the finding that consumers are willing to pay the premium for eco-friendly products even when they are told that the products do not have any health benefits [32].

Most consumers are not prepared to pay a premium for eco-labeled alternatives and, by habit [27], [51], choose conventional products instead [52]–[56]. As shown here, from a seller's perspective, the highest average price for a product can be extracted if the consumer is told about the eco-label whilst they have already said that they prefer that product, regardless of the customers' attitudes toward sustainable consumption (Experiment 2). This technique could potentially be used to by-pass consumer habit and promote purchase of eco-labeled products in those who would normally be unwilling to pay a premium for those alternatives. Exploring this possibility is a target for future research, especially in field settings wherein the participants ‘true’ willingness to pay are measured rather than the hypothetical ‘stated’ willingness to pay that was measured here, as stated willingness to pay may not be entirely consistent with how consumers behave in ‘reality’. Moreover, low sustainability consumers appear to base their willingness to pay on taste to a greater degree than their high sustainability counterparts, perhaps because they are generally less altruistic. If this is the case, one way to promote purchase of eco-labeled products by those individuals could be to use more egoistically centered labels (e.g., health benefits).

A final conclusion concerns a potential non-separability between objective and subjective product characteristics. Our results show that the timing by which an individual learns about the subjective (non-tangible) characteristics of a product affects his or her evaluation of the objective (tangible) characteristics. This may have implications for economic models of demand estimation in which goods are treated as a bundle of characteristics.

### Conclusion

Eco labels not only promote a willingness to pay more for the product but they also lead to a more favorable perceptual experience of it. Understanding the psychological mechanisms that underpin the eco-label effect and how to modulate its magnitude could potentially be a key to promote sustainable consumer behavior.
